# Editorial: Non-coding RNA elements as regulators of host–pathogen interactions

**DOI:** 10.3389/fgene.2024.1374636

**Published:** 2024-03-05

**Authors:** Margarida Gama-Carvalho, Nham Tran

**Affiliations:** ^1^ BioISI—Biosystems & Integrative Sciences Institute, Faculty of Sciences, University of Lisboa, Lisboa, Portugal; ^2^ School of Biomedical Engineering, Faculty of Engineering and Information Technology, University of Technology Sydney, Ultimo, NSW, Australia

**Keywords:** ncRNA, host–pathogen interaction, gene expression, virus, parasites, RNA modifications, circular RNA, innate immunity

Microbes such as viruses, bacteria, and protozoa often cause infections in a variety of organisms, with the course and outcome of these infections being shaped by intricate interactions between the host and pathogen. These interactions include the host’s immune responses, both innate and adaptive, which are met with countermeasures by the pathogen. A crucial aspect of these interactions is the regulated expression of genes, where non-coding RNA (ncRNA) and RNA-binding proteins are key players. The role of ncRNA in gene expression is universal across all life forms, evident from the regulatory sequences in mRNA molecules to various types of ncRNA molecules, including small ∼20 nucleotide-long elements involved in target recognition and larger catalytic ribozymes (Morris and Mattick, 2014).

The growing use of high-throughput sequencing technologies has brought to light the large numbers of genes producing non-coding transcripts across all types of genomes. These include, among many others, microRNAs (miRNAs), PIWI-interacting RNAs (piRNAs), and the more recently discovered circular RNAs (circRNAs), the three classes of ncRNAs that are specifically discussed by the articles integrating this Research Topic. A major hurdle that remains once a non-coding transcript is detected is to establish that it has a function rather than being a product of random transcription or degradation. Although these molecules are found across all types of organisms and microbes, underscoring the pervasiveness of RNA-dependent regulation, their functional relevance is questioned in several specific cases, when the biogenesis pathway remains unclear, and the relative abundance is low. Such issues tend to become more significant in complex biological systems like host–pathogen interactions. In this Research Topic, Ruivinho and Gama-Carvalho discuss how the identification of small ncRNAs (sncRNAs) encoded by RNA viruses and discovery of their role in the regulation of infectious processes have been hampered by both methodological limitations and conventional thinking.

Increasing research efforts on RNA virus-derived sncRNAs during the past few years, more recently fueled by the SARS-CoV-2 pandemic, are leading to significant paradigm changes regarding their biogenesis, prevalence, and functional relevance in the context of host–pathogen interactions, underscoring their widespread presence across all virus groups.

Beyond their centrality in the regulation of gene expression programs in this dynamic context, RNA molecules are one of the key molecular features used by the host’s innate immune systems to detect the presence of pathogens (Liu and Gack, 2020). Interestingly, microRNAs are key regulators of eukaryotic gene expression that are believed to have evolved from systems that may have originally acted to detect invading nucleic acids (Koonin, 2017). In addition to detecting microbe infection, such systems have been crucial at the evolutionary level to ensure the protection of genomes against replicating nucleic acids like transposons and retrotransposons, particularly in the germline cells of multicellular organisms. This control is achieved by the activity of ribonucleoprotein complexes involving the highly conserved PIWI subfamily of Argonaute proteins and a special class of sncRNAs called piRNAs. In another manuscript in this Research Topic, Horjales et al. reviewed evidence for the presence of non-canonical complexes of PIWI proteins and miscellaneous piRNAs in non-germinal cells of the *Trypanosoma cruzi* and *Toxoplasma gondii* parasites, where they play a role in the regulation of gene expression programs and host–parasite interactions. Indeed, these authors had previously reported the presence of a *T. cruzi* PIWI-like complex as the cargo of secreted extracellular vesicles relevant in intercellular communication and host infection (Garcia-Silva et al., 2014).

The secretion of regulatory RNA molecules by parasites with potential impact on host biology is also reported in this Research Topic in an original research article by Minkler et al., who reported the identification of thousands of distinct circRNA molecules from adult female stages of the gastrointestinal parasitic nematode *Ascaris suum*. These results are in line with our own previous work on the worm parasite *Fasciola hepatica*, demonstrating the secretion of miRNAs that hijack the host macrophage machinery to modulate early innate immune responses (Tran et al., 2021).

Together, these observations expand the known toolkit of pathogens and parasites to modulate host biology and improve the outcome of their infectious process. The use of secreted RNA molecules by pathogens to interfere with host cells clearly highlights the relevance of mounting innate defense systems that are able to effectively detect and destroy them. In this context, RNA editing and the use of different types of nucleotide modification are increasingly being implicated in both the distinction between self and non-self RNA molecules and in counteracting pathogen responses. Ribeiro et al. discussed how these systems play a central role in the context of viral infections, highlighting the potential of targeting the RNA epitranscriptome for novel antiviral therapies.

This Research Topic highlights ncRNA’s critical and pervasive role in regulating infectious processes across various host–pathogen pairs. It aims to encourage researchers to explore similar regulatory pathways in different organisms, potentially uncovering unique mechanisms that not only deepen our understanding of these complex processes but may also pave the way for novel therapeutic approaches.
